# Potential Role of High‐Intensity Interval Training‐Induced Increase in Humanin Levels for the Management of Type 2 Diabetes

**DOI:** 10.1111/jcmm.70396

**Published:** 2025-02-12

**Authors:** Afsaneh Soltany, Farhad Daryanoosh, Firouzeh Gholampour, Najmeh Sadat Hosseini, Kayvan Khoramipour

**Affiliations:** ^1^ Department of Biology, College of Science Shiraz University Shiraz Iran; ^2^ Department of Sports Sciences, Faculty of Educational Sciences and Psychology Shiraz University Shiraz Iran; ^3^ Physiology and Neuroscience Research Center, Institute of Physiology and Pharmacology Kerman University of Medical Sciences Kerman Iran; ^4^ i+HeALTH Strategic Research Group, Department of Health Sciences Miguel de Cervantes European University (UEMC) Valladolid Spain

**Keywords:** apoptosis, HIIT, humanin, inflammation, oxidative stress

## Abstract

This study investigated the effect of 8 weeks of high‐intensity interval training (HIIT) on oxidative stress, inflammation, and apoptosis in rats with type 2 diabetes (T2D), focusing on the role of the Humanin (HN). In this study, 28 male Wistar rats were assigned to one of four groups: healthy control (CO), diabetes control (T2D), exercise (EX), and diabetes + exercise (T2D + EX). After diabetes induction (2‐month high‐fat diet and injection of 35 mg/kg streptozotocin), the animals in the EX and T2D + EX groups underwent an 8‐week HIIT protocol (4–10, interval of 80%–100% of maximum speed). HOMA‐IR, fasting blood glucose, and HN levels were measured in the serum. The expression of HN, Bax, Bcl‐2, CAT, GPx, MDA, TNFα, and IL‐10 was measured in the soleus muscle. Our results showed that the serum level of HN and the muscle levels of IL‐10, SOD, CAT, and Bax were higher in the T2D + EX group than in the T2D group. However, the HOMA‐IR index and the muscle levels of MDA, TNFα, and Bcl‐2 were lower in the T2D + EX group than in the T2D group. Muscle levels of HN and GPx showed no significant difference between the T2D + EX and T2D groups. The result of Pearson analysis showed a significant correlation between HN and MDA, SOD, Bax and Bcl‐2. This study provides evidence that there is a correlation between serum Humanin levels and HIIT. HIIT benefits T2D rats by reducing inflammation and oxidative stress. Given Humanin's established involvement in inflammation and oxidative stress, it is possible that the benefits of HIIT on T2D rats are mediated by humanin.

## 1 Introduction

Type 2 diabetes (T2D) is a metabolic disorder and one of the leading causes of mortality worldwide [1]. Insulin resistance (IR), a hallmark of T2D, leads to hyperglycaemia and subsequent oxidative stress and inflammation, ultimately resulting in apoptosis [2, 3, 4]. Reactive oxygen species (ROS) play a key role in this process by causing oligomerisation of B‐cell lymphoma protein 2 and (Bcl‐2)‐associated X (Bax), which form pores in the mitochondrial outer membrane and release cytochrome c into the cytosol, leading to caspase‐3 activation and apoptosis [5, 6]. Additionally, oxidative stress stimulates the production of inflammatory cytokines such as tumour necrosis factor‐alpha (TNF‐α), further exacerbating IR [7, 8, 9, 10]. TNF‐α also reduces insulin receptor substrate 1 (IRS‐1) serine phosphorylation and inhibits insulin receptor tyrosine kinase activity, which reduces cell responsiveness to insulin [11, 12].

Humanin (HN) is a mitochondrial‐derived polypeptide that has been shown to have a protective effect on inflammation, apoptosis and the oxidative stress response caused by diabetes [13, 14]. HN was initially discovered for its neuroprotective effects [15]. HN is produced by various tissues, such as the brain, liver, heart, testes, and skeletal muscles [16, 17, 18]. HN is secreted into the blood circulation and delivered to target cells via HN receptors [19], where it binds to membrane receptors and intracellular molecules, inducing their function [20]. The serum and skeletal muscle levels of HN have been observed to decrease in individuals with T2D [13, 21]. This has prompted researchers to explore strategies for elevating HN levels, aiming to mitigate the detrimental effects associated with diabetes. HN activates the phosphatidylinositol 3‐kinase (PI3K)/protein kinase B (AKT) signalling pathway, inhibiting the proapoptotic proteins Bax, Bak, and caspase‐9, which can improve IR and glucose consumption and reduce gluconeogenesis [17, 22].

Exercise has been proposed as a non‐pharmacological treatment that raises HN levels. It has been reported that endurance exercise increases HN levels by 39%, which remain elevated 3 h after exercise [23]. Furthermore, high‐intensity interval training (HIIT) increased plasma and muscle HN levels [24]. No studies have investigated the effects of exercise‐induced Humanin on oxidative stress, inflammation, and apoptosis in T2D. Therefore, in this study, we investigated the effect of 8 weeks of HIIT on oxidative stress, inflammation, and apoptosis in rats with T2D, focusing on the role of the HN.

## 2 Materials and Methods

### 2.1 Animals

Twenty‐eight male Wistar rats weighing 200–250 g were purchased and kept in standard cages with a 12 h/12 h light/dark cycle at an ambient temperature of 24°C ± 1°C and a humidity of 50% ± 4%. Throughout the experiment, the animals had unlimited access to food and water. The Ethics Committee of KUMS approved the study protocol prior to any experiments being carried out (Ethics Approval Code: IR.KMU.REC.1399.503). These animals were randomly divided into four groups: healthy control (CO), exercise (EX), diabetes (T2D), and diabetes + exercise (T2D + EX) (7 rats per group).

### 2.2 Induction of Type 2 Diabetes

Wistar rats were fed a high‐fat diet (i.e., 60% fat, 20% protein, and 20% carbohydrate) for 8 weeks before being injected with 35 mg/kg streptozotocin (STZ) [25]. When fasting blood glucose (FBG) was more than 300 mg/dL 72 h after STZ injection, diabetes was confirmed [26].

### 2.3 Training Protocols

High‐intensity interval training (HIIT) was performed in this study for 8 weeks, 5 days per week, according to the K1 protocol as described in detail in our previous publication [27, 28, 29, 30]. The rats were acclimated to the treadmill for 5 days at an 8 m/min speed and a 0° inclination for 10 min. To determine the rat's maximum speed (*V*_max_), each rat was forced to run on a treadmill (6 m/min, 2 min, incline 0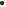), and the treadmill speed was gradually increased (every 2 min by 2 m/min) until exhaustion. The final effort of each rat was considered the maximum speed [30]. Warm‐up and cool‐down exercises were performed for 5 min at 40%–50% *V*_max_ at the start and end of each training session. The details of the training protocol are shown in Table 1.

**TABLE 1:** Training protocol.

Total duration of the training	Speed during rest interval (%*V*_max_)	Duration of rest interval (min)	Speed during high‐intensity interval (%*V*_max_)	Duration of high‐intensity interval (min)	Interval numbers	Week
12	50	1	80	2	4	1
12	50	1	85	2	4	2
18	50	1	85	2	6	3
18	50	1	90	2	6	4
24	50	1	90	2	8	5
24	50	1	95	2	8	6
30	50	1	95	2	10	7
30	50	1	100	2	10	8

### 2.4 Sampling

Forty‐eight hours after the last training session, the animals were anaesthetised via intraperitoneal injection of ketamine (80 mg/kg) and xylazine (10 mg/kg). Blood samples were then collected from the animal's hearts after a 12‐h fasting period, and soleus muscles were harvested for further analysis. The collected blood samples were allowed to stand at room temperature for 30 min before being subjected to centrifugation at 1000×*g* for 20 min at 4°C. Following centrifugation, the resulting serum samples were stored at a temperature of −80°C to preserve their integrity for subsequent examination. Moreover, the harvested soleus muscle was thoroughly washed with a PBS solution comprising 1.37 M NaCl, 27 mM KCl, 100 mM Na_2_HPO_4_, and 18 mM KH_2_PO_4_ at a pH of 7.4. To perform homogenisation, an ultrasonic homogeniser was used in combination with RIPA buffer (150 mM NaCl, 1.0% IGEPAL CA‐630, 0.5% sodium deoxycholate, 0.1% SDS, 50 mM Tris, pH 8.0) supplemented with a protease inhibitor to ensure sample stability during the process. The homogenisation procedure was carried out on ice to maintain the sample's structural integrity and to prevent any potential degradation.

Following homogenisation, the mixture was centrifuged at 4°C for 20 min. The resulting supernatant, containing the extracted components of interest, was carefully preserved at a temperature of −80°C, ensuring optimal conditions for subsequent analysis and experimentation.

### 2.5 Western Blot

The soleus muscle was analysed using Western blotting to determine Bax (Cat. No: (*N* = 19) sc‐7480), Bcl2 (Cat. No: (N‐19): sc‐492) and HN (Cat. No: PA1‐4359) levels. Lysis buffer was used to lyse the tissues, and the homogenate was centrifuged in an Eppendorf 5415 R centrifuge at a temperature of 4°C and 12,000 rpm for 10 min. The supernatant was extracted and stored at −20°C. The proteins were separated via electrophoresis via SDS‐PAGE, after which the proteins were transferred to PVDF membranes. After that, the membrane was mixed with 2% skim milk in TBS for 1 h. In the next step, the membrane was incubated with the primary antibody, and after the addition of the secondary antibody and washing steps, the proteins were visualised using an enhanced chemiluminescence (ECL) kit and imaged. Finally, the qualitative data were converted into quantitative data by ImageJ software. β‐Actin was used as a control.

### 2.6 Elisa

Serum levels of HN (Cat. No: DEIA10628), muscle TNF‐α (Karmania pars gene Co, Kerman, Iran, CN: KPG‐R TNF‐α Ka), muscle IL‐10 (Karmania pars gene Co, Kerman, Iran, CN: KPG‐RIL10 Ka) and insulin (Rat ELISA Kit, Eastbiopharm) were measured using the Elisa kit.

### 2.7 Antioxidant System Markers

Muscle SOD activity was determined using the method of Paoletti and Mocali [31]. This method uses superoxide radicals produced upon hypoxanthine oxidation by xanthine oxidase to react with 2‐(4‐iodophenyl)‐3‐(4‐nitrophenol)‐5‐phenyltetrazolium chloride (I.N.T.) and form a red formazan dye. SOD activity was measured by the degree to which this reaction was inhibited. One unit of SOD is the amount of enzyme needed to dismutase 50% of the superoxide radicals.

Muscle GPx activity was evaluated using the method described by Paglia and Valentine [32]. GPx catalyses the oxidation of glutathione. In the presence of glutathione reductase and NADPH, oxidised glutathione is converted to its reduced form. NADPH extinction was read at 412 nm to indicate glutathione peroxidase activity.

Muscle Catalase activity was determined based on the ability of catalase to decompose hydrogen peroxide (H_2_O_2_) into water and oxygen. The rate of disintegration of hydrogen peroxide into water and oxygen is proportional to the catalase concentration.

Muscle levels of Malonildialdehyde (MDA) were measured using the thiobarbituric acid method. To measure the MDA concentration, 20 μL of homogenised muscle tissue supernatant, a reaction mixture containing 150 μL of thiobarbituric acid, 20 μL of sodium dodecyl sulfate (SDS), 150 μL of 20% acetic acid (pH = 3.5) and 60 μL of distilled water were mixed. The resulting mixture was heated at 90°C for 45 min, and after cooling to room temperature, it was centrifuged at 10,000×*g* for 10 min to obtain a clear solution. Then, the absorbance was recorded at 532 nm. The amount of MDA was calculated based on the standard curve of tetramethoxypropane.

### 2.8 FBG, Insulin, Insulin Resistance and Sensitivity Indices

The insulin resistance index was calculated using the homeostasis model assessment‐insulin resistance (HOMA‐IR) [33] by measuring fasting insulin and glucose levels, and to estimate the level of insulin sensitivity, the insulin sensitivity index (QUIUKI: Quantitative insulin sensitivity check index) was calculated [29].

HOMA‐IR = (Fasting Insulin (μU/ml) × Fasting Glucose (mmol/l))/22.5.

QUICKI = 1/[log (Fasting Insulin) + log (Fasting Glucose)].

### 2.9 Statistical Analysis

GraphPad Prism v.9 software was used for statistical analysis. The Shapiro test was used to determine the normality of the distribution of the data. One‐way ANOVA and Tukey's post hoc test were used to investigate the significant differences between the research groups. In addition, we conducted Pearson correlation analysis to study the correlation between the measured variables with serum concentration of HN. The data are presented as the mean ± SD, and a significance level less than 0.05 was used.

## 3 Results

### 3.1 Effect of 8 Weeks of HIIT on Serum and Soleus Muscle HN

**FIGURE 1:**
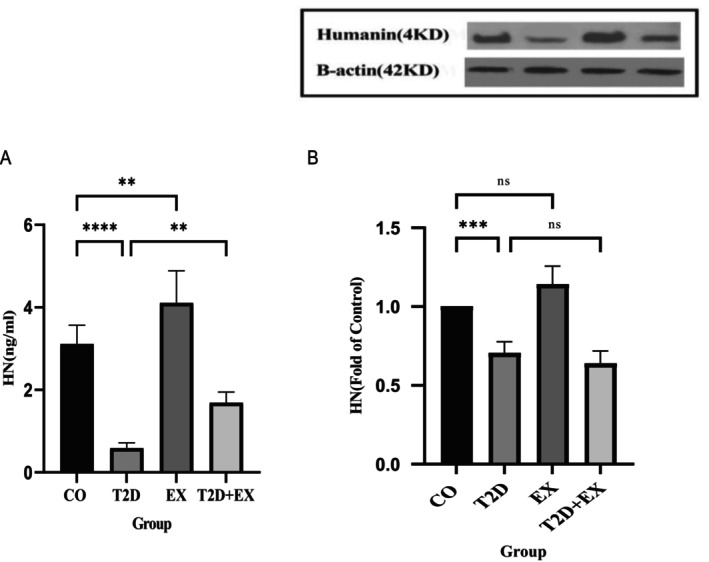
HN levels in the serum (A) and soleus muscle (B) after 8 weeks of HIIT. The data are presented as the mean ± SD (*n* = 7 in each group). CO, control; T2D, type 2 diabetes; EX, exercise; T2D + EX: type 2 diabetes+ exercise. ****p < 0.0001, ***p < 0.001, **p < 0.01, ns, no significant difference.

Our results showed that serum HN levels (*F*_3,20_ = 65. 41, *p* < 0.0001) and soleus muscle (*F*_3,16_ = 45.25, *p* < 0.0001) were significantly different between the groups. HN levels in the serum and soleus muscle were lower significantly in the T2D group compared to those in the CO group (*p* < 0.0001 and *p* < 0.001, respectively); however, after 8 weeks of HIIT, serum HN levels (*p* < 0.01) was higher. Compared with those in the T2D group, the serum levels of HN in the T2D + EX group were greater (*p* < 0.01). However, this difference was not significant in the soleus muscle (Figure 1), suggesting that HN can enter the circulation from other tissues like muscle [24, 34], resulting in a further increase in the serum HN concentration.

### 3.2 Effect of 8 Weeks of HIIT on CAT, GPx, SOD, and MDA Levels in Soleus Muscle

**FIGURE 2:**
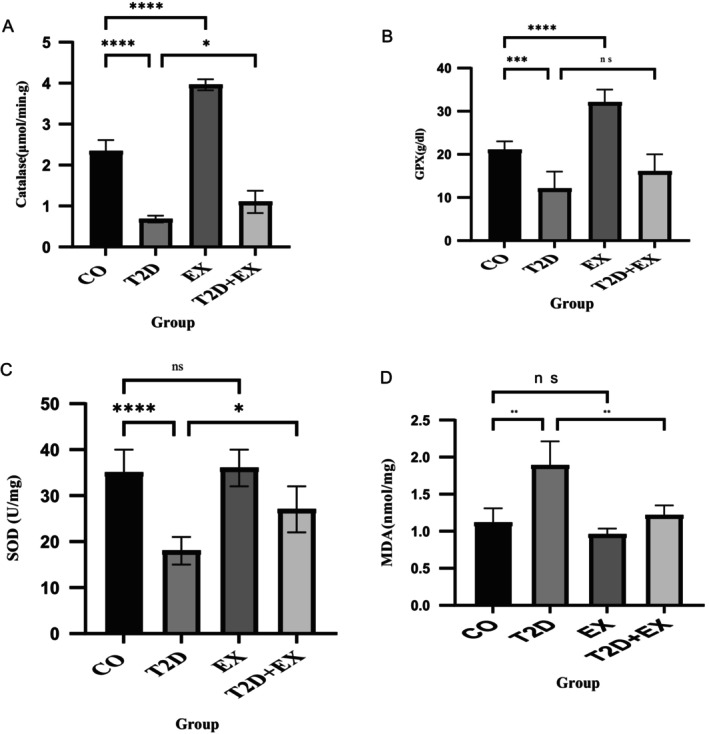
Effect of 8 weeks of HIIT on CAT (A), GPx (B), SOD (C), and MDA (D) levels in muscle tissue. The data are presented as the mean ± SD (*n* = 7 in each group). CO, control; T2D, type 2 diabetes; EX, exercise; T2D + EX, type 2 diabetes + exercise. ****p < 0.0001, ***p < 0.001, **p < 0.01, *p < 0.05, ns, no significant difference.

Our findings indicate that the levels of CAT (*F*_3,16_ = 250. 8, *p* < 0.0001), GPx (*F*_3,20_ = 39.96, *p* < 0.0001), and SOD (*F*_3,20_ = 22.40, *p* < 0.0001) were significantly lower in the T2D group than in the CO group. However, compared with those in the T2D group, the CAT and SOD levels in the T2D + EX group were significantly greater (*p* < 0.05) (Figure 2A–C). These results suggest that HN may have the potential to reverse the decreases in SOD and CAT levels observed in the T2D group.

Moreover, our results showed that MDA levels were significantly different between the groups (*F*_3,24_ = 27.52, *p* < 0.0001). MDA levels in the T2D group were greater than those in the CO group (*p* < 0.01) but lower than those in the T2D + EX group (*p* < 0.01) (Figure 2D).

### 3.3 Effect of 8 Weeks of HIIT on IL‐10 and TNF‐α in Soleus Muscle

**FIGURE 3:**
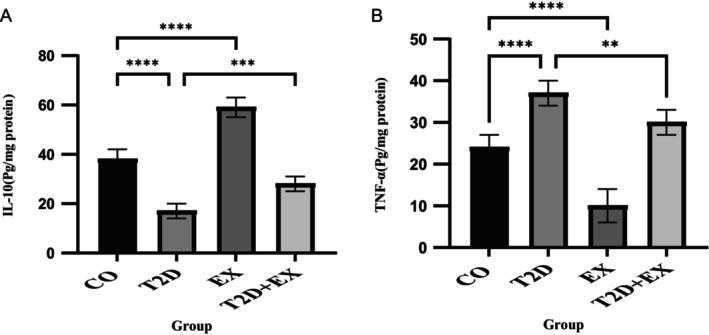
Effect of 8 weeks of HIIT on IL‐10 (A) and TNF‐α (B) levels. The data are presented as the mean ± SD (*n* = 7 in each group). CO, control; T2D, type 2 diabetes; EX, exercise; T2D + EX, type 2 diabetes + exercise. ****p < 0.0001, ***p < 0.001, **p < 0.01.

Our results showed that the muscle levels of IL‐10 (*F*_3,20_ = 153. 1, *p* < 0.0001) and TNF‐α (*F*_3,20_ = 73.44, *p* < 0.0001) were different between the groups. IL‐10 levels were lower and greater in the T2D (*p* < 0.0001) and EX (*p* < 0.0001) groups, respectively, than in the CO group. Conversely, the muscle TNF‐α concentration was greater in the T2D group (*p* < 0.0001) but lower in the EX (*p* < 0.0001) group than in the CO group. Furthermore, the T2D + EX group exhibited a significantly greater level of IL‐10 (*p* < 0.001) but a lower level of TNF‐α (*p* < 0.01) than did the T2D group (Figure 3).

### 3.4 Effect of 8 Weeks of HIIT on Bax and Bcl‐2 in Soleus Muscle

**FIGURE 4:**
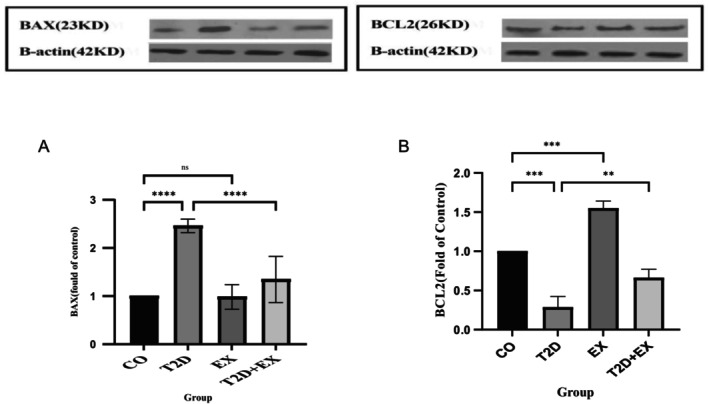
Effect of 8 weeks of HIIT on the expression of Bax (A) and Bcl‐2 (B) in the soleus muscle. The data are presented as the mean ± SD of *n* = 7 in each group. CO, control; T2D, type 2 diabetes; EX, exercise; T2D + EX, type 2 diabetes + exercise. ****p < 0.0001, ***p < 0.001, **p < 0.01, ns, no significant difference.

Our results revealed that the expression of Bax (*F*_3,20_ = 36.71, *p* < 0.0001) and Bcl‐2 (*F*_3,16_ = 19. 73, *p* < 0.0001) were significantly different between the groups. Bax levels were greater in the T2D group than in the CO (*p* < 0.0001) and T2D + EX (*p* < 0.0001) groups. However, Bcl2 levels were significantly lower in the T2D group than in the CO (*p* < 0.0001) and T2D + EX (*p* < 0.05) groups (Figure 4).

### 3.5 Fasting Blood Glucose (FBG)

**FIGURE 5:**
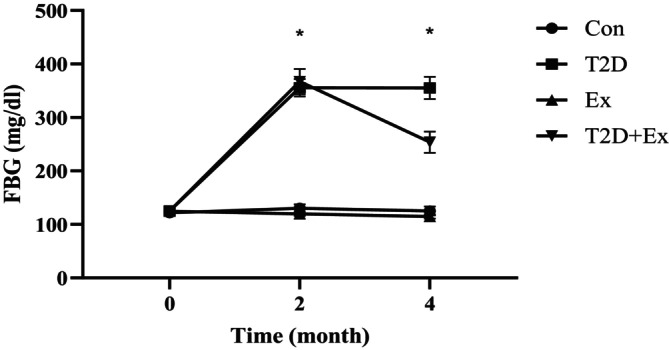
FBG levels (mean ± SD) before starting the intervention (month 0), after diabetes induction (2 months of high‐fat diet and STZ injection) (month 2), and 48 h after the last training session (month 4) in all groups. FBG, fasting blood glucose; CO, control; T2D, type 2 diabetes (STZ injected); EX, exercise; T2D + EX, type 2 diabetic + exercise. *A significant difference between T2D patients and T2D + EX patients.

We evaluated FBG levels to validate our approach in inducing diabetes. The findings indicated a substantial increase in FBG levels after inducing diabetes (via a high‐fat diet and STZ injection over 2 months) at month 2 compared to the baseline measurements taken at month 0 for both the T2D and T2D + EX groups (*p* = 0.000). There were no notable differences between these groups. Furthermore, HIIT significantly reduced blood glucose levels (*p* = 0.000) (Figure 5).

### 3.6 Insulin Sensitivity Indices

**FIGURE 6:**
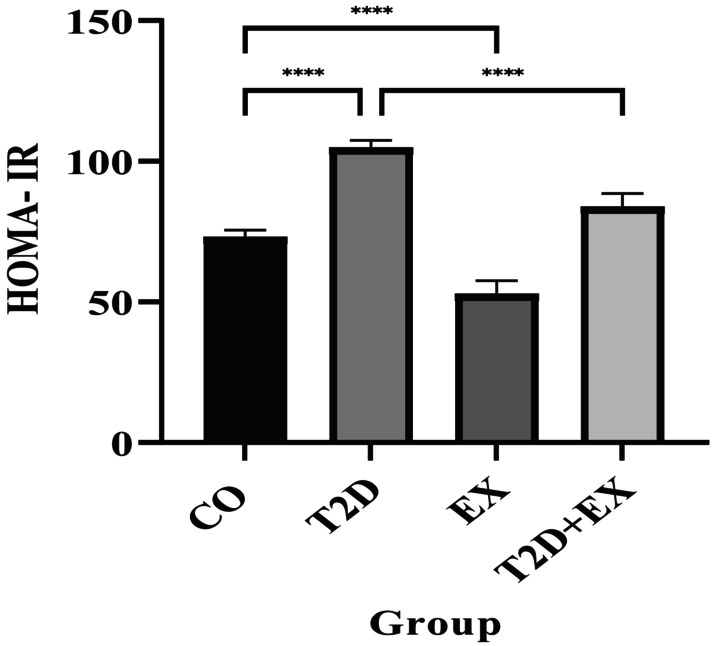
HOMA‐IR: Homeostatic Model Assessment for Insulin Resistance. Data are presented as the mean ± SD, *n* = 7 in each group. CO, control; T2D, type 2 diabetes; EX, exercise; T2D + EX, type 2 diabetes + exercise. ****p < 0.0001.

To investigate the potential impact of exercise on insulin sensitivity, we examined the HOMA‐IR score. The results revealed that HOMA‐IR was significantly different between the groups (*F*_3,16_ = 159.1, *p* < 0.0001). It was greater in the T2D group than in the CO (*p* < 0.0001) and T2D + EX (*p* < 0.0001) groups (Figure 6).

### 3.7 Correlation Analysis

The result of Pearson analysis showed a significant correlation between HN and MDA (*p* ≤ 0.001), SOD (*p* ≤ 0.04), Bax (*p* ≤ 0.005), and Bcl‐2 (*p* ≤ 0.01) but not the others (Table S1).

## 4 Discussion

T2D stands out as one of the prevailing diseases in the world. It has been documented that a robust association exists between hyperglycemia, oxidative stress induced by hyperglycemia, inflammation, and the advancement and escalation of T2D [9]. It has been documented that HN displays cytoprotective properties, by alleviating oxidative stress, apoptosis, and inflammatory reactions [35]. The aim of this study was to investigate the effect of 8 weeks of HIIT on oxidative stress, inflammation, and apoptosis in rats with T2D, focusing on the role of the HN. Our results revealed higher plasma levels of HN and lower levels of markers associated with oxidative stress (i.e., MDA), apoptosis (i.e., Bax), and inflammation (i.e., TNF‐α) in the soleus muscle following HIIT in T2D rats. Furthermore, the levels of antioxidants (i.e., SOD and CAT), anti‐inflammatory agents (i.e., IL‐10), and antiapoptotic agents (i.e., Bcl‐2) were greater in the T2D + EX group than in the T2D group. However, our correlation analysis showed a correlation for just SOD, MDA, Bax, and Bcl‐2 with serum HN concentration. As well as a notable decrease in blood glucose levels was observed following 8 weeks HIIT in diabetic rats.

The results of our investigation revealed that induction of T2D led to a significant decrease in Humanin levels in both muscle and plasma. Studies indicate that serum HN concentrations are lower in T2D [21]. Maria et al. [36] also mentioned that patients with type 1 diabetes exhibit lower levels of Humanin. Our results suggested that the serum level of HN, as opposed to its concentration in the soleus muscle, exhibited a positive reactionary trend following HIIT in T2D rats. The elevation in the serum HN concentration might stem from its secretion by various organs, such as the brain, liver, heart, testes, and skeletal muscle. Nevertheless, the response of skeletal muscle appears to be contingent upon its specific fiber type. This discrepancy becomes evident when comparing our findings to those of Gidlund et al. [37], who documented escalated levels of HN in the vastus lateralis muscle of middle‐aged men (54 ± 7 years) with prediabetic conditions following 12 weeks of resistance training. However, our study did not observe a parallel increase in the HN concentration within the soleus muscle. This disparity could be attributed to the rigorous nature of our training protocol, which is characterised by high intensity (80%–100% of *V*_max_) and may have resulted in limited engagement of the predominantly slow‐twitch soleus muscle. Furthermore, HN has been found to interact with certain proteins within cells so it could be that the binding is obscuring the increase. This interaction could be another reason for the lack of a significant increase in the soleus muscle level of HN. The lack of a significant increase in skeletal muscle Humanin levels following 12 weeks endurance type Nordic walking training aligns with our findings [37].

An increase in the serum HN concentration following exercise is thought to be a reaction to the stress that exercise imposes on mitochondria [24]. This stress ultimately contributes to an enhanced defence against harmful oxidative factors and a reduction in oxidative stress [38]. In line with these findings, our findings revealed a decrease in MDA levels and an increase in the levels of antioxidant enzymes, such as CAT and SOD, however, a correlation was just seen for MDA and SOD with serum HN concentration. A range of potential explanations for this phenomenon are that HN can activate a crucial transcription factor called nuclear factor erythroid 2‐related factor 2 (Nrf2), which plays a pivotal role in protecting cells against oxidative stress [39]. Furthermore, in accordance with prior studies, HN reduces the oxidative stress via increasing AMPK phosphorylation [40], reducing expression of NADPH oxidase 2 [41], and inhibiting mTOR [42]. On the other hand, the lack of a correlation between HN and CAT could suggest that the action of HN may not directly influence hydrogen peroxide breakdown or that its effects may be more linked to the early stages of oxidative stress (e.g., scavenging superoxide radicals) rather than hydrogen peroxide processing [43, 44] In addition, MDA is a marker of lipid peroxidation, a consequence of oxidative stress and could correlate with SOD, an enzyme that defends against oxidative damage by neutralising superoxide radicals. The fact that HN serum levels correlate with both SOD and MDA may indicate that HN plays a protective role in cellular damage related to lipid peroxidation, enhancing the activity of SOD in the process. This could be particularly relevant in diabetes, where oxidative stress is elevated.

Reducing oxidative stress can potentially decrease inflammation [45]. Moreover, functioning as an anti‐inflammatory mediator within immune responses, HN plays a role in mitigating inflammation by engaging with the gp130 subunit of the IL‐6 receptor [39]. This interaction consequently leads to a reduction in the generation of proinflammatory cytokines such as TNF‐α. Additionally, Humanin triggered by exercise has been observed to notably elevate IL‐6 levels [35]. The role of IL‐6 can be proinflammatory or anti‐inflammatory, contingent on the specific cells secreting it. While its secretion by T cells and macrophages tends to induce an inflammatory effect, its secretion by muscle cells transforms it into an anti‐inflammatory cytokine [46]. This capacity inhibits the production of TNF‐α and enhances the production of IL‐10. Our findings align with this phenomenon, as we noted increased IL‐10 levels and decreased TNF‐α level in the soleus muscle. However, our correlation analysis did not show a significant relationship between HN and either TNF‐α or IL‐10. It is possible that the anti‐inflammatory effects of HN apply through alternative pathways or cytokines (such as IL‐6, IL‐1β or NF‐κB signalling), which may not directly correlate with TNF‐α or IL‐10 levels.

Diminishing oxidative stress can also contribute to a reduction in apoptosis [47]. The decrease in the expression of Bax and the concurrent increase in Bcl‐2 levels imply that HN might promote cell survival by hindering the process of apoptosis. The significant correlation between HN and Bax and Bcl‐2 further reinforces this hypothesis.

In the context of diabetes, HN extends protection against apoptosis prompted by cytokines [48]. This safeguarding effect is achieved through the interaction of HN with the proapoptotic protein Bax. This interaction inhibits Bax translocation to the mitochondria and subsequently curtails the initiation of apoptosis via Bax. This modulation occurs either through engagement with the FPRL‐1 receptor or through direct interaction with Bax [49]. As a result, it is highly plausible that the increase in HN induced by exercise could effectively regulate the protective influence of exercise against mitochondria‐mediated apoptosis.

In conclusion, our study demonstrated that HIIT can potentially regulate HN levels in diabetic rats. This regulation may lead to an experimental therapeutic approach for improving inflammation, oxidative stress, and apoptosis.

### 4.1 Study Limitations

One limitation of this study is the absence of baseline measurements for each experimental group prior to the training intervention. Due to the invasive nature of muscle tissue collection, obtaining baseline data would have required sacrificing animals before the HIIT protocol, limiting our ability to complete the intervention with the same subjects. Instead, we conducted a controlled comparison among groups post‐intervention. However, we acknowledge that incorporating a separate baseline group would have strengthened our findings by providing clearer insights into the relative impact of HIIT.

Another limitation is the inability to directly assess the activation of key signalling pathways related to Humanin, such as PI3K/AKT, due to financial constraints. This restricts our understanding of the precise molecular mechanisms by which HIIT influences Humanin levels and its role in modulating oxidative stress and inflammation in Type 2 diabetes.

While we measured TNF‐α and IL‐10 as indices of inflammation, the lack of correlation between HN and these variables suggests that HN may influence other inflammatory mediators not captured in our analysis.

Additionally, the study did not directly assess the response of fast‐twitch muscles, which could provide a clearer understanding of the role of HN in HIIT. Future research should focus on fast‐twitch muscle fibres to further elucidate the production and systemic effects of HN and its interaction with slow‐twitch muscles, such as the soleus.

## Author Contributions

**Afsaneh Soltany:** conceptualization (equal), formal analysis (equal), investigation (lead), software (equal), visualization (equal), writing – original draft (lead). **Farhad Daryanoosh:** project administration (equal), supervision (lead), writing – review and editing (supporting). **Firouzeh Gholampour:** writing – review and editing (equal). **Najmeh Sadat Hosseini:** writing – original draft (supporting). **Kayvan Khoramipour:** supervision, funding acquisition (equal), project administration (equal), supervision (lead), writing – review and editing (equal).

## Disclosure

The authors declare that there are no relationships or activities that might bias, or be perceived to bias, their work.

## Ethics Statement

The ethics committee of Kerman University of Medical Sciences approved all procedures conducted in this study under the authority of the Project Licence (IR.KMU.REC.1399.503). This study was conducted in accordance with the Animal Rights and the National Institutes of Health Guide for the Care and Use of Laboratory Animals (publication no. 85‐23, revised 1985). Additionally, all the authors confirm that all the procedures, including protocols and experiments, were conducted in full compliance with the applicable ARRIVE guidelines.

## Consent

The authors have nothing to report.

## Conflicts of Interest

The authors declare no conflicts of interest. All the coauthors have read and agreed with the contents of the manuscript, and there are no financial interests to report. We certify that the submission is original work and is not under review for publication elsewhere.

## Supporting information


Appendix S1.

## Data Availability

The data that support the findings of this study are available at University of Shiraz.
