# Supplementary material for: Potential Role of High‐Intensity Interval Training‐Induced Increase in Humanin Levels for the Management of Type 2 Diabetes
Source: J Cell Mol Med. 2025 Feb 12;29(3):e70396. doi: 10.1111/jcmm.70396 (PMC11815479; doi:10.1111/jcmm.70396)
Supplement: Supplementary file 1 — Appendix S1. [file JCMM-29-e70396-s001.docx]

Supplementary table 1. Correlation between HNS and other variables

|  | HNS  vs.BAX | HNS  vs. BCL2 | HNS  vs. MDA | HNS  vs. IL10 | HNS  vs. GPX | HNS  vs. SOD | HNS  vs. Catalase | HNS  vs. TNF |
| --- | --- | --- | --- | --- | --- | --- | --- | --- |
| r | 0.2369 | 0.5896 | 0.4569 | -0.1909 | -0.1880 | 0.4569 | 0.3095 | 0.1888 |
| R squared | 0.3698 | 0.6359 | 0.5632 | 0.03643 | 0.03535 | 0.3268 | 0.09577 | 0.03564 |
| P (two-tailed) | 0.005* | 0.01* | 0.001* | 0.5524 | 0.5585 | 0.04* | 0.1843 | 0.5568 |

*Shows significant difference
